# Reply: An inverse association between tumour size and overdiagnosis may explain the results by Bucchi *et al*

**DOI:** 10.1038/sj.bjc.6602583

**Published:** 2005-04-26

**Authors:** L Bucchi, A Barchielli, A Ravaioli, M Federico, V De Lisi, S Ferretti, E Paci, M Vettorazzi, S Patriarca, A Frigerio, E Buiatti, and the SCREENREG Working Group

**Affiliations:** 1 Romagna Cancer Registry, Forlì, Italy; 2 Epidemiology Unit, ASL 10, Firenze, Italy; 3 Modena Cancer Registry, Modena, Italy; 4 Parma Cancer Registry, Parma, Italy; 5 Ferrara Cancer Registry, Ferrara, Italy; 6 Tuscany Cancer Registry/Epidemiology Unit, CSPO, Firenze, Italy; 7 Veneto Cancer Registry, Padova, Italy; 8 Piedmont Cancer Registry, CPO Piemonte, Torino, Italy; 9 First Screening Centre, Torino, Italy; 10 Tuscany Regional Health Agency, Firenze, Italy

**Sir**,

Thank you for the opportunity to reply to the issues raised in the letter by Dr Zahl regarding our paper (Bucchi *et al*, 2005).

The study focused on the potential for biological progression of screen-detected (SD) breast cancers. In brief, we assumed the following: (1) the average risk of axillary lymph node metastases is lower for SD cases than clinical cases; (2) nodal status is the product of biological aggressiveness and chronological age of the disease; (3) tumour size is a proxy indicator of chronological age; and (4) specifically for SD tumours, size is an indicator of duration of preclinical phase. The hypothesis was that the relative protection of SD tumours from the risk of nodal involvement (i.e. their relative biological indolence) decreases with increasing size. We built a multiple regression model that included a term for the detection-mode-by-tumour-size interaction. This was strong and significant. The relative risk of nodal involvement for SD tumours increased from 0.05 in the 2–7 mm size category to 0.95 in the 18–22 mm category, while decreasing again among the relatively small number of SD tumours of larger size. We found only one plausible and coherent interpretation of such a pattern, namely (1) the biological aggressiveness of most breast cancers increases during the preclinical phase; (2) only a small subset of screen-detected cancers have a relatively stable biological indolence; and (3) they become apparent only among the few large-sized tumours detected at first screen.

Dr Zahl suggests that the length time bias and its extreme form, overdiagnosis, may explain those observations. In particular, he disagrees with our statement that ‘…no published data support an inverse association between overdiagnosis and tumour size…’. Length bias and overdiagnosis in mammography screening are not under debate. Their potential magnitude, however, is ill-defined. The probability of slow-growing SD tumours being classified as overdiagnosed is closely dependent on duration of follow-up. In the greater part of published estimates, the ratio of the lifetime cumulative number of cases in a screened population to the number in a control population varies between zero (Olsen *et al*, 2003) and 20% (Wald *et al*, 1994). When we wrote the paper we did not know yet of the work by Zahl *et al* (2004), who reported an excess incidence as high as 50%. It is beyond the scope of this brief note to address the methodological concerns that have been raised regarding the design of that study. In brief, as stated by Duffy in an accompanying Editorial (2005), it seems unlikely that the length bias could entirely explain the interaction effect observed in our data.

In any case, the magnitude of length bias (and of overdiagnosis, if any) is not the key to the interpretation of our findings. A much more critical question is: are slow-growing tumours equally distributed by size? In fact, length bias may explain the observed downward trend in the relative risk of nodal metastases only if it is assumed that the prevalence of slow-growing SD cancers decreases with increasing tumour size. The problem is more complicated than it appears. As cancers overdiagnosed are SD, and SD cancers are relatively small, it is conceivable to conclude that there is an ecological association between overdiagnosis and small lesions. However, it does not follow that the prevalence of overdiagnosis is inversely related to tumour size. If the rate of onset of new slow-growing tumours is constant, the prevalence of such cases is not expected to be greater among SD cancers 2–7 mm in size than it is in the 18–22 mm category. In fact, we are not aware of any reported observation suggesting this association in a formal fashion.

Assuming that the onset of new slow-growing tumours follows a constant rate, the next question is: what may cause the prevalence of indolent SD tumours to decrease with size? As Dr Zahl will certainly remember, we took into consideration the hypothesis that the natural prevalence of indolent cancers among those SD is altered by mammography itself, that is, that mammography sensitivity decreases selectively for such lesions as their size increases. Such a hypothesis is clearly implausible: not only has it never been demonstrated, the issue has never even been raised. We also discussed another potential type of overdiagnosis, namely, the histological misinterpretation of benign lesions as malignant. In fact, it is not reasonable to assume that such a shortcoming occurs more often among SD cancers than clinical cancers of the same size. Nor it is likely that histology evaluation is less accurate in the breast surgery reference hospitals involved in screening. If the onset of slow-growing tumours is constant and these ‘exogenous’ factors are not at play, it seems to us that only one conclusion remains: the prevalence of indolent lesions decreases because of tumour progression.

We did not cite the investigation by Joensuu *et al* (2004) because its end point, tumour size-specific survival of SD cancers compared with clinical cancers, was not closely relevant to the design of our own. We acknowledge, however, that findings in that study were consistent with the relative biological indolence of SD tumours being a constant feature. The only point we make is that Joensuu *et al* categorized tumour size into conventional 10-mm categories, whereas our case series could only be categorized into clusters around multiples of 5 mm (Figure 1 in the paper). It is worth noting that our conclusion that the biological aggressiveness of most breast cancers increases as they develop in size relies critically on the key observation that SD cancers 18–22 mm in diameter had the same risk of lymph node involvement as clinical cancers. If we had used the conventional cutoff value of 20 mm, these cases would have been equally diluted among those in the lower and higher size categories, both with fewer nodal metastases than clinical cancers of the same size. Incidentally, we agree with the remark made by Duffy (2005) in the accompanying Editorial. The practical implications of the observed frequency distribution of tumour size go far beyond our speculations about the impact of mammography on the natural history of breast cancer.

We thank Dr Zahl for his comments. As a closing consideration, however, we would like to emphasise that a thorough debate on the meaning of our findings is rather premature. Prior to our study, only a few univariate observations had suggested that tumour size interacts with detection mode in determining the risk of lymph node metastases (Anderson *et al*, 1991; Ernst *et al*, 2002). The only previous study formally aimed at demonstrating this effect (Tabar *et al*, 1987) had negative results. As pointed out by Duffy (2005), our findings are unique and await confirmation.
